# Proteomic analysis of exosome-like vesicles from *Fasciola gigantica* adult worm provides support for new vaccine targets against fascioliasis

**DOI:** 10.1186/s13071-023-05659-7

**Published:** 2023-02-10

**Authors:** Zhao-An Sheng, Cui-Lan Wu, Dong-Ying Wang, Shu-Hong Zhong, Xi Yang, Guo-Shun Rao, Hao Peng, Shi-Wen Feng, Jun Li, Wei-Yi Huang, Hong-Lin Luo

**Affiliations:** 1 Institute of Oncology, Guangxi Academy of Medical Sciences, Nanning, Guangxi People’s Republic of China; 2 Guangxi Colleges and Universities Key Laboratory of Prevention and Control for Animal Disease, College of Animal Science and Technology, Guangxi University, Nanning, People’s Republic of China; 3 Guangxi Key Laboratory of Veterinary Biotechnology, Guangxi Veterinary Research Institute, Nanning, Guangxi People’s Republic of China; 4 Department of Pathogenic Biology, Jining Medical University, Shandong, People’s Republic of China; 5 Yuxi Animal Disease Prevention and Control Center, Yuxi, People’s Republic of China; 6 Key Laboratory of China (Guangxi)-ASEAN Cross-Border Animal Disease Prevention and Control, Ministry of Agriculture and Rural Affairs of China, Nanning, Guangxi People’s Republic of China

**Keywords:** *Fasciola gigantica*, Extracellular vesicles, Exosome-like vesicles, Fascioliasis, Proteomics, Vaccine

## Abstract

**Background:**

Extracellular vesicles (EVs) released by helminths play an important role in parasite-host communication. However, little is known about the characteristics and contents of the EVs of *Fasciola gigantica*, a parasitic flatworm that causes tropical fascioliasis. A better understanding of EVs released by *F. gigantica* will help elucidate the mechanism of *F. gigantica*-host interaction and facilitate the search for new vaccine candidates for the control and treatment of fascioliasis.

**Methods:**

Two different populations of EVs (15k EVs and 100k EVs) were purified from adult *F. gigantica* culture media by ultracentrifugation. The morphology and size of the purified EVs were determined by transmission electron microscopy (TEM) and by the Zetasizer Nano ZSP high performance particle characterization system. With the aim of identifying diagnostic markers or potential vaccine candidates, proteins within the isolated 100k EVs were analyzed using mass spectrometry-based proteomics (LC–MS/MS). Mice were then vaccinated with excretory/secretory products (ESPs; depleted of EVs), 15k EVs, 100k EVs and recombinant* F. gigantica* heat shock protein 70 (rFg-HSP70) combined with alum adjuvant followed by challenge infection with *F. gigantica* metacercariae. Fluke recovery and antibody levels were used as measures of vaccine protection.

**Results:**

TEM analysis and nanoparticle tracking analysis indicated the successful isolation of two subpopulations of EVs (15k EVs and 100k EVs) from adult *F. gigantica* culture supernatants using differential centrifugation. A total of 755 proteins were identified in the 100k EVs. Exosome biogenesis or vesicle trafficking proteins, ESCRT (endosomal sorting complex required for transport) pathway proteins and exosome markers, heat shock proteins and 14-3-3 proteins were identified in the 100k EVs. These results indicate that the isolated 100k EVs were exosome-like vesicles. The functions of the identified proteins may be associated with immune regulation, immune evasion and virulence. Mice immunized with *F. gigantica* ESPs, 15k EVs, 100k EVs and rFg-HSP70 exhibited a reduction in fluke burden of 67.90%, 60.38%, 37.73% and 56.6%, respectively, compared with the adjuvant control group. The vaccination of mice with *F. gigantica* 100k EVs, 15k EVs, ESP and rFg-HSP70 induced significant production of specific immunoglobulins in sera, namely IgG, IgG1 and IgG2a.

**Conclusion:**

The results of this study suggest that proteins within the exosome-like vesicles of *F. gigantica* have immunomodulatory, immune evasion and virulence functions. This knowledge may lead to new strategies for immunotherapy, vaccination and the diagnosis of fascioliasis.

**Graphical Abstract:**

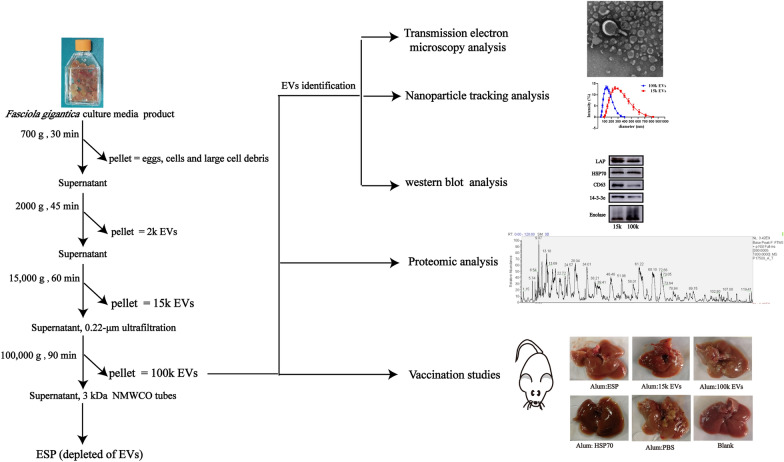

**Supplementary Information:**

The online version contains supplementary material available at 10.1186/s13071-023-05659-7.

## Background

The digenetic trematode, *Fasciola gigantica*, is an important food-borne zoonotic parasite with a worldwide distribution. Infection with this parasitic flatworm can cause lack of appetite, diarrhea, liver damage, anemia, reduced weight gain and reduced milk production [1]. These symptoms result in substantial economic losses, especially in developing countries, and threaten human health [2]. *Fasciola gigantica* has developed adaptations to establish chronic infections through the suppression of host immunity [3]. Specifically, *F. gigantica* infections generally induce a non-protective T helper 2/regulatory T cells (Th2/Treg) immune response and suppression of the protection provided by T helper 1 (Th1) immune responses. This response could be initiated by the release of excretory/secretory products (ESPs) [4]. The ESPs released by *F. gigantica* can directly regulate the differentiation of macrophages, dendritic cells (DCs) and T cells, and they have a modulating effect on DCs, thereby promoting downstream Th2/Treg responses [5, 6]. *Fasciola gigantica* ESPs also induce macrophages toward an alternatively activated macrophage (M2 cells) phenotype, which promotes Th2/Treg responses [7, 8].

Helminth-derived extracellular vesicles (EVs) play an essential role in parasite-host communication by transferring parasite material to the host [9]. They can be released by the gastrointestinal tract or protonephridial system of the parasite or shed directly from the parasite’s tegument and converged into ESPs [10, 11]. EVs are small membrane-enclosed nanoparticles that are released by many organisms, including parasitic helminths and their hosts. They contain different proteins, lipids, messenger RNAs and non-coding RNAs [12, 13]. EVs released by a broad spectrum of helminths (e.g., *Fasciola hepatica* [14], *Opisthorchis viverrini* [15]*, Heligmosomoides polygyrus* [16], *Schistosoma japonicum* [17], *Echinococcus multilocularis* [18] and *Echinococcus granulosus* [19]) can modulate the host immune response or transportation of virulence factors [20]. For example, EVs isolated from the ESPs of *H. polygyrus* can be assimilated by macrophages and inhibit macrophage activation through downregulation of pro-inflammatory tumor necrosis factor alpha (TNF-α) and interleukin-6 (IL-6) [21]. *Opisthorchis viverrini* has a strong association with cholangiocarcinoma, and the internalization of *O. viverrini* EVs by cholangiocytes promotes cell proliferation, IL-6 secretion and induction of protein expression associated with cancer [15]. EVs released by *S. japonicum* can be taken up by immune cells (including macrophages) and transferred as microRNA cargo into recipient cells for the suppression of TNF-α production [17]. *Echinococcus multilocularis* EVs can inhibit nitric oxide (NO) and pro-inflammatory cytokines produced by activated RAW264.7 macrophages [18]*.* EVs isolated from *E. granulosus* protoscoleces and metacestodes from the supernatants of cultures suppressed the maturation of DCs and the antigen presentation pathway, which may play a role in immunomodulation [19].

The molecular composition of EVs secreted by *F. gigantica* may be involved in immune regulation of the host and *F. gigantica*–host communication. For a better understanding of the relationship between *F. gigantica* and its host, we used differential ultracentrifugation to isolate two types of EVs (15k EVs and 100k EVs) from the supernatants of adult *F. gigantica* cultures. We also used mass spectrometry (MS)-based proteomics (liquid chromatography [LC]–MS/MS) analyses of proteins within the isolated 100k EVs to demonstrate that the 100k EVs contained several proteins related to immune regulation, immune evasion and virulence. These proteins are possible diagnostic markers and vaccine candidates. We also show that *F. gigantica* secretes ESPs, 15k EVs and 100k EVs that induce protective immunity to infection.

## Methods

### Animals

Female ICR mice aged 5–7 weeks were purchased from Hunan SJT Laboratory Animal Co., Ltd. (Changshang, China) and maintained under pathogen-free conditions in the laboratory animal room of the Parasitology Unit, Guangxi University, China.

### Preparation of ESPs

Adult *F. gigantica* parasites were obtained from the gallbladders of freshly killed swamp buffaloes at local abattoirs. Adult parasites were washed three times with phosphate-buffered saline (PBS), then cultured in PBS (1 worm/1 ml PBS) at 37 °C for 60 min to remove host contaminants (e.g., bile, liver tissue and blood) from the guts. The parasites were then incubated in RPMI 1640 culture medium (GIBCO, Grand Island, NY, USA) containing 0.1% glucose, 100 U penicillin and 100 μg/ml streptomycin (Solarbio, Beijing, China), at 1 worm/ml culture medium for 5 h at 37 °C. The supernatant was collected and used for the next step.

### EV isolation and purification

Extracellular vesicles were isolated from *F. gigantica* culture media according to the differential centrifugation protocol reported by Marcilla et al. [22] with some modifications. Briefly, the parasite culture media was centrifuged at 300 *g* for 15 min and then at 700 *g* for 30 min to remove eggs, cells and large cell debris. The supernatant was then centrifuged at 2000 *g* for 45 min at 4 °C, and the resulting supernatant was centrifuged at 15,000 *g* for 60 min at 4 °C to obtain 15k EVs (mainly microvesicles). ESP supernatants were then filtered through a 0.22-μm ultrafiltration membrane and the filtered supernatants centrifuged at 100,000 *g* for 90 min at 4 °C to pellet the 100k EVs (exosome-like EVs). Both the 15k and 100k EV pellets were washed twice with a large volume of PBS at the same high speed. The resulting supernatant was concentrated using Amicon® Ultra-15 3 kDa molecular weight cut-off (NMWCO) tubes (MilliporeSigma, Burlington, MA, USA) at 5000 *g *for 4 °C and then washed 3 times with PBS. Protein concentrations of ESP supernatants (depleted of EVs) consist of filtered samples and the 3k permeate. Protein contents were quantified by the bicinchoninic acid assay (BCA) kit (CWBIO, Beijing, China) and then stored at − 80 °C for further studies.

### Transmission electron microscopy analysis of EV samples

The 15k pelleted EVs and 100k pelleted EVs were positioned on formvar-coated copper grids (Zhongjingkeyi Technology, Beijing, China) and stained with 2% phosphotungstic acid (Solarbi) for 1 min and at room temperature. EVs on the grids were imaged using a Hitachi-H7700 transmission microscope (Hitachi, Tokyo, Japan) and exposed at 100 kV.

### Nanoparticle tracking analysis

To measure the size distribution of particles in the EV samples, nanoparticle tracking analysis (NTA) was carried out using the Zetasizer Nano ZSP high performance particle characterization system (Malvern Instruments, Malvern, UK) to capture and analyze the data. EVs were diluted in PBS and then loaded into the sample chamber. For each sample, three measurements were performed and the data were analyzed.

### Proteomic analysis of *F. gigantica* 100k EVs

Proteomic analysis was done on *F. gigantica* 100k EVs, and two samples that had been independently separated were assayed. The 100k EV protein samples were digested with trypsin as described by Wu and Liu [23], with some modifications. Briefly, approximately 60 μg of the 100k EV sample was first reduced with 100 mM dithiothreitol (DTT) and then incubated in a boiling water bath for 5 min, following which 200 μl of UA buffer (8 M urea, 150 mM Tris–HCl, pH 8.0) was added and the solution mixed. The mixture was transferred to an ultrafiltration device (10 kDa) for centrifugation, and the supernatant was removed. Then 100 μl iodoacetamine (IAA) buffer (100 mM IAA in UA buffer) was added to the sample pellet and the mixture shocked for 1 min. The samples were incubated for 30 min in darkness at room temperature, following which 100 μl of UA buffer was added and centrifuged; this procedure was repeated twice. Then 100 μl of 25 mM of NH_4_HCO_3_ was added and the mixture centrifuged; this procedure was repeated twice. The proteins were then incubated with 5 μg of trypsin in 40 μl of 100 mM NH_4_HCO_3_ at 37 °C for 18 h, followed by centrifugation. Finally, 40 μl of 25 mM of NH_4_HCO_3_ was added, and the mixture was centrifuged and acidified. The digestion products were collected for subsequent analysis. According to the quantitative results, 3 µg of the digested peptides was analyzed by LC–MS/MS (Thermo Fisher Scientific, Waltham, MA, USA). Digested peptides pre-equilibrated with buffer A (0.1% v/v formic acid aqueous solution) were added to a 5-μm C18 EASY column (2 × 100 μm; Thermo Fisher Scientific) and separated with a linear gradient of buffer B (0.1% v/v formic acid in 84% v/v acetonitrile aqueous solution) on a 3 μm-C18 EASY column (75 μm × 100 mm 3-μm C18 column; Thermo Fisher Scientific). The Easy nLC system (Thermo Fisher Scientific) was used to deliver buffer A and buffer B with a linear gradient of 0–55% (0–110 min), 55–100% (110–115 min) and maintained at 100% (115–120 min), at a flow rate of 300 nl/min. The MS scan data were acquired using the Q-Exactive spectrometer (Thermo Fisher Scientific), and the 20 most abundant precursor ions from the survey scan (300–1800 *m*/*z*) were selected for higher-energy collisional dissociation fragmentation. The automatic gain control (AGC) target was set to 3e6, maximum inject time (IT) was 50 ms and dynamic exclusion duration was 60.0 s. Using predictive automatic gain control, scans were acquired at a resolution of 70,000 at* m*/*z* 200 with a dynamic exclusion duration of 60 s, and the target value was determined for full scans. The resolution for the high-energy collisional dissociation spectra was set to 17,500 at* m*/*z* 200, with an isolation window at 2 *m*/*z*. The normalized collision energy was set at 27 eV, and the underfill ratio was defined as 0.1%.

The FASTA database was from *F. hepatica* (consisting of 26,283 entries, downloaded on 10 Jan 2020) and from *F. gigantica* (consisting of 13,094 entries) obtained from UniProt (https://www.uniprot.org/) were used for the analysis of the EV mass spectrometry data through Proteome Discoverer 2.2 (Thermo Fisher Scientific). Only proteins with at least three peptides were considered to be positively identified.

### Protein expression and purification of recombinant* F. gigantica* heat shock protein 70

The full-length sequence of heat shock protein 70 (HSP70) (GenBank: ABS52703.1) of *F. gigantica* was obtained from the GenBank database. To produce HSP70, the *F. gigantica* HSP70 gene sequence was first synthesized; following codon optimization, the gene sequence cloned into a pET28a ( +) vector using* Bam*HI and* Xho*I cloning sites. The expression pET28a(+) vector was transformed into *Escherichia coli* BL21 (DE3) competent cells and induced with 0.1 mM isopropylthio-β-galactoside (IPTG) (Solarbio) at 30 °C for 8 h. Recombinant * F. gigantica* HSP70 (rFg-HSP70) was purified by affinity chromatography using a His-tag Protein Purification Kit (Beyotime, Haimen, Jiangsu, China). New Zealand White rabbits were immunized with rFg-HSP70 to produce polyclonal antibodies against rFg-HSP70, using the method described by Anuracpreeda et al. [24].

### Sodium dodecyl sulfate-polyacrylamide gel electrophoresis and western blot analysis

The 15k and 100k EVs (in 20-ug samples) were loaded onto a 15% sodium dodecyl sulfate-polyacrylamide gel electrophoresis (SDS-PAGE) gel for separation. The protein products were transferred to 0.45-μm polyvinylidene difluoride membranes (MilliporeSigma). The membranes were blocked with blocking solution (PBST: PBS, 0.05% Tween-20, 5% non-fat powdered milk) for 4 h at room temperature, followed by overnight incubation at 4 °C with rabbit anti-human CD63 monoclonal antibody (dilution 1:500; Abcam, Cambridge, UK) and rabbit polyclonal antibodies raised against *F. gigantica* leucine amino peptidase (LAP), HSP70 and 14-3-3 epsilon (dilution 1:200). Antibodies were prepared previously in our laboratory [25]. The membranes were then washed 5 times for 10 min each time with PBST, followed by incubation for 4 h at 4 °C with the secondary goat anti-mouse immunoglobulin G (IgG; (dilution 1:5000; Abcam) antibody conjugated with horseradish peroxidase (HRP). After five washes of 10 min each with PBST for chemiluminescence imaging with a BeyoECL Plus kit (Beyotime), the membranes were visualized using the ImageQuant LAS 500 imager (Cytiva, Marlborough, MA, USA).

### Vaccination studies on experimental animals and vaccination protocol

Forty 7-week-old female ICR mice were randomly divided into five groups (8 mice/group): (i) a non-immunized and uninfected group (Blank group); (ii) an infected group immunized with PBS + alum adjuvant (Thermo Fisher Scientific, Rockford, IL, USA) (Alum + PBS group); (iii) an infected group immunized with 200 μg of ESP (depleted of EVs) + alum adjuvant (Alum + ESP group); (4) an infected group immunized with 200 μg of 15k EVs + alum adjuvant (Alum + 15k EVs group); (v) an infected group immunized with 200 μg of 100k EVs + alum adjuvant (Alum + 100k EVs group); and (vi) an infected group immunized with 200 μg of rFg-HSP70 + alum adjuvant (Alum + rHSP70 group). Mice were maintained in the laboratory animal room of Parasitology Unit, Guangxi University, China. All animal experimental procedures were performed according to the National Institutes of Health guide for the care and use of laboratory animals (NIH Publication No. 8023, revised 1978). Mice were kept in steel cages in an air-conditioned room at 23 ± 2 °C, under a 12:12-h (L/D) photoperiod and 50–60% relative humidity. Vaccines were injected intraperitoneally on days 0, 14 and 28. Treated mice were challenged with 15 *F. gigantica* metacercariae via the oral route of administration, on day 42. At 28 day post-challenge, mice were sacrificed using CO_2_, and blood samples were collected for serum by heart puncture. The peritoneal cavities were opened and washed with PBS. Whole livers were collected to determine the number of *F. gigantica* flukes (Fig. 4).

### Worm recovery and percentage protection

The percentage reduction of *F. gigantica* worm recovery was calculated as previously described [26]. Worm reduction (%) = (A − B)/A) × 100%, where A = worm burden immunized with PBS + alum adjuvant group and B = worm burden in the challenged immunized group.

### Antiparasite IgG1 and IgG2a/c ELISAs

Specific IgG, IgG1 and IgG2a against *F. gigantica* 15k EVs, *F. gigantica* 100k EVs or *F. gigantica* ESPs were carried out by indirect enzyme-linked immunosorbent assay (ELISA) as described previously [3]. The serum IgG, IgG1, and IgG2a antibody response against *F. gigantica* 15k EVs, 100k EVs and ESPs was measured, in triplicate, for each group by indirect ELISA.

### Statistical analyses

Results are presented as the means ± standard error of the mean (SEM), and analyses were performed using GraphPad Prism software version 6.01 (GraphPad Software Inc., San Diego, CA, USA). Statistical analysis was evaluated by one-way analysis of variance (ANOVA) followed by Tukey’s post hoc test or Student’s t-test. *P* < 0.05 indicated statistical significance (*P < 0.05, **P < 0.01, ***P < 0.001).

## Results

### Characterization of 15k EVs and 100k EVs isolated from *F. gigantica* ESPs

**Fig. 1:**
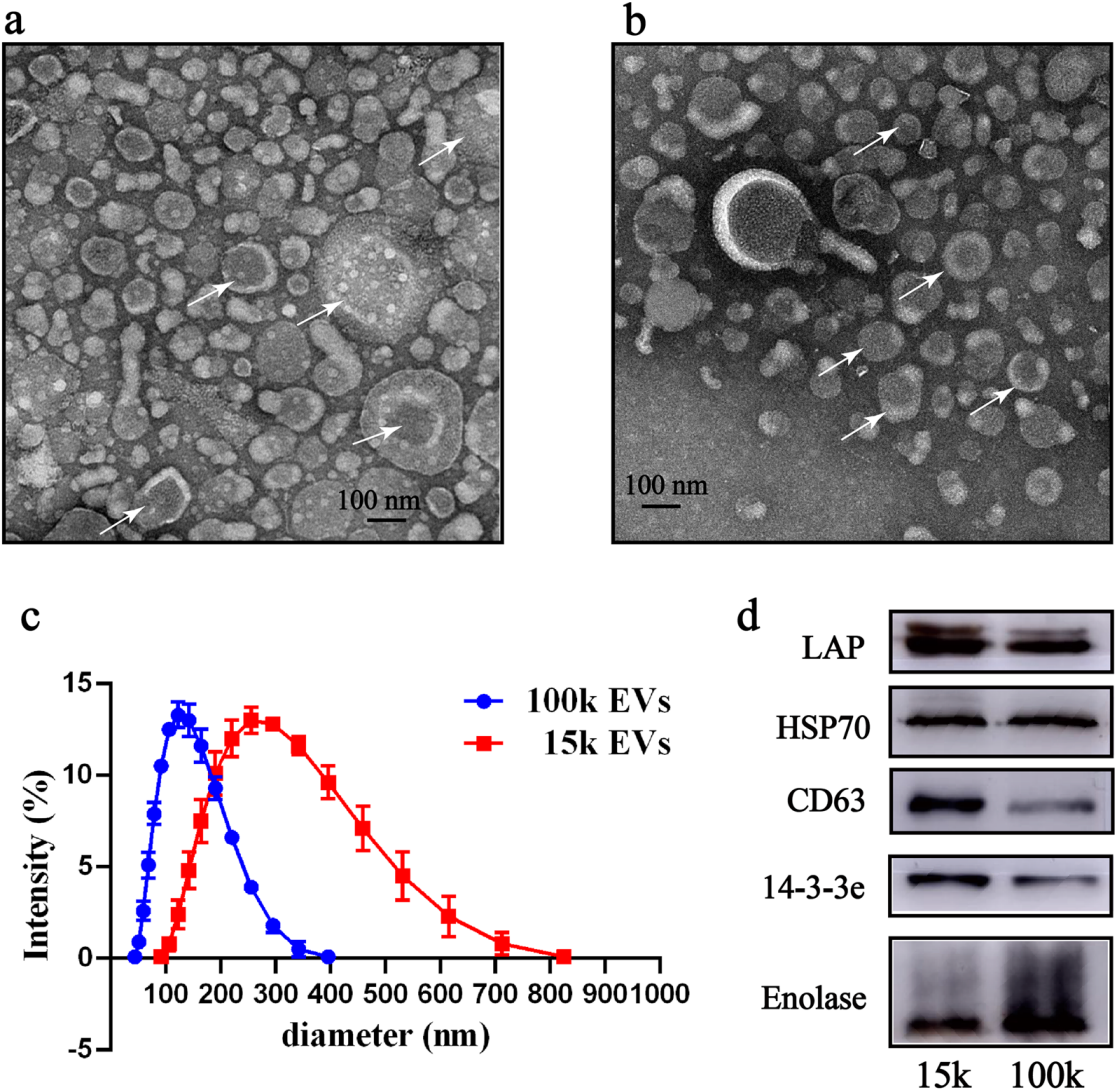
Identification of different *Fasciola gigantica* extracellular vesicles (EVs). **a**, **b** Morphological characterization by TEM by TEM of 15k EVs and 100k EVs released by adult *F. gigantica*. Arrowheads indicate EVs stained with 2% phosphotungstic acid. **c** Diameter distribution of the purified 15k EVs and 100k EVs from adult *F. gigantica*. The results represent data from three independent *F. gigantica* 15k EV and 100k EV preparations analyzed by the Zetasizer Nano ZSP high performance particle characterization system. **d** Western blot analysis of *F. gigantica* 15k EVs and 100k EVs using antibodies against *F. gigantica* leucine amino peptidase (LAP), heat shock protein 70 (HSP70), 14-3-3epsilon (14-3-3e), enolase and human CD63. TEM, Transmission electron microscopy

We used the BCA kit to determine the relative contributions of the 15k EVs, 100k EVs and ESP supernatant to the total amount of protein secreted by adult *F*. *gigantica*. We found that *F*. *gigantica* secreted total protein at a rate of 64.3 μg/fluke/h (100%). A secretion rate of 2.85 μg/fluke/h (4.4%) was associated with the 15k EVs and a secretion rate of 1.3 μg/fluke/h (2.0%) was associated with the 100k EVs; however, the majority of soluble proteins (> 90%) remained in the ESP. The results from the transmission electron microscopy (TEM) and NTA studies confirmed that we had successfully isolated two different types of EVs from the *F*. *gigantica* culture media. Pellets from the 15,000 *g* centrifugation ranged from 91.28 nm to 825 nm in diameter, with most vesicles (> 74%) larger than 200 nm (Fig. 1c) and with typical characteristics of microvesicles (Fig. 1a). Pellets from the 100,000 *g* centrifugation were composed of vesicles ranging in diameter from 43.82 to396.1 nm, with most vesicles (> 86%) being < 200 nm in diameter (Fig. 1c) and with cup-shaped structures (Fig. 1b).

### Proteomic analysis of *F. gigantica*-derived 100k EVs

A total of 705 proteins were identified (≥ 3 matched unique peptides) from *F. gigantica*-derived 100k EVs (Additional file 1: Table S1). Among the identified proteins, the most abundant proteins were dynein heavy chain 1 cytosolic, otoferlin, myoferlin, programmed cell death 6-interacting protein, vacuolar protein-sorting-associated protein 4, receptor mediated endocytosis, serine/threonine-protein kinase PAK and leucine amino peptidase (Table 1). Many proteins that have been observed in exosomes, such as exosome biogenesis or vesicle trafficking proteins (annexin, tetraspanin family members, acid sphingomyelinase, myoferlin, otoferlin, charged multivesicular body protein, Rab family members, vacuolar protein sorting 26, syntenin), endosomal sorting complex required for transport (ESCRT; including ESCRT-II complex subunit, charged multivesicular body proteins, protein IST1, syntenin), heat shock proteins and 14-3-3 [11, 27], were identified in *F. gigantica*-derived 100k EVs (Additional file 1: Table S1). Among these proteins, HSP70, CD63, 14-3-3, annexin and glyceraldehyde-3-phosphate dehydrogenase (GAPDH), which are typically found in helminth exosomes like EVs, were identified (Fig. 1d; Table 1). The identification of these proteins indicates the successful isolation of exosome-like vesicles from adult *F. gigantica* culture supernatants. Several proteins identified in the 100k EVs have already been characterized as possible vaccine candidates against fascioliasis, including cathepsin L [28], leucine aminopeptidase [29], 14-3-3 [30], fatty acid binding protein (FABP) [31], phosphoglycerate kinase [32], cathepsin B [33], and tetraspanin 2 (-TSP2) [34], but with different protective efficacies.

**Table 1:** Top 50 proteins identified by mass spectrometry from 100k extracellular vesicles isolated from adult *Fasciola gigantica* culture supernatants

**Accession no. **	**Description**	**Unique peptides**	**Molecular weight (kDa)**	**pI**
A0A504YKS2	Dynein heavy chain 1 cytosolic	66	540.7	6.44
A0A4E0S279	Otoferlin	59	242.6	6.05
A0A504YEK9	Transient receptor potential cation channel subfamily Mmember 2	50	200.2	7.23
A0A4E0RXP6	Myoferlin	37	179.1	6.3
A0A4E0RX62	Programmed cell death 6-interacting protein	37	86.3	5.81
A0A504Z6J5	Receptor Mediated Endocytosis	37	62.2	6.98
A0A504Z0R7	Vacuolar protein-sorting-associated protein 4	37	49	8.53
A0A504YKR3	Serine/threonine-protein kinase PAK	36	54.2	8.03
C6KGZ9	Leucine amino peptidase	32	56.3	7.62
A0A504YLZ2	Uncharacterized protein	32	113.3	6.77
B1NI97	Heat shock protein 70	29	70.7	5.74
A0A4E0R767	Putative cytosolic ca2 + -dependent cysteine protease calpain	29	88.9	7.17
A0A4E0RJT9	Calpain-B	28	85.7	5.6
A0A504Y4G4	Long-chain-fatty-acid-CoA ligase	28	76.6	8.43
A0A2H1BY53	Phosphopyruvate hydratase	27	61.3	7.4
A0A2H1C3P3	14-3-3 protein	26	28	5.3
A0A504YH77	Malic enzyme	26	63.7	8.18
A0A504YP52	Annexin	26	101.3	6.58
A0A504YZD9	Translation elongation factor 2	26	94.6	6.93
A0A504YRB3	BRO1 domain and CAAX motif containing	25	48.6	7.31
A0A504YS13	Hepatocyte growth factor-regulated tyrosine kinase substrate	24	85.6	6.65
A0A504YKC9	Fatty-acid amide hydrolase 1	24	69.6	8.22
A0A504YB52	Galectin	24	123.6	6.86
A0A504Z9J2	Serine/threonine kinase	23	65.7	9.45
A0A504YJX4	Autophagy-related protein 9	23	95.5	8.5
A0A4E0RG70	Rab GDP dissociation inhibitor	22	51.6	6.6
A0A504Z761	Severin	22	43.1	6.14
A0A504Z3L5	Heat shock protein kDa	22	69.8	7.56
A0A504YE87	C2 domain	22	134	6.3
A0A504Y6B9	Uncharacterized protein	21	36.8	9.23
A0A504YSL7	L-plastin	21	73.8	6.07
A0A504Z4Z3	DnaJ like protein subfamily A member 1	21	44.8	6.84
A0A504YVC1	Niemann-Pick C1 protein	21	152.5	5.88
A0A504YUZ5	Synaptosomal-associated protein 25-A	20	27.1	6.54
A0A4E0S0V7	Putative ankyrin repeat-containing	20	88	6.58
A0A504X7M0	Leukotriene A-4 hydrolase	20	77.6	7.97
A0A504YNB9	Charged multivesicular body protein	20	68.4	5.52
A0A4E0S286	Pcd6 interacting protein	19	204.6	6.84
A0A504YZP0	Carboxylic ester hydrolase	19	78.9	6.57
A0A504YHJ1	T-cell immunomodulatory protein	19	70.3	5.1
A0A504YNI3	STAM-binding protein A	19	44	6.58
A0A504Y9P3	Major vault protein isoform X1	19	99.7	5.48
A0A504YQE4	Putative vacuolar protein sorting 26 vps26	18	60.7	5.91
A0A2H1CH07	Rab-protein 8	18	25.3	6.09
A0A504Y734	Putative spindle pole body protein	18	39.4	5.06
A0A504YNN3	Vacuolar protein sorting-associated protein VTA1	18	31.8	5.92
A0A504YUZ4	Cyclin-Y	18	51.8	7.84
Q45UT3	Phosphoglycerate kinase	18	47	8.87
A0A504YH94	Band 3 anion transport protein	18	113.6	8.43
A0A4E0R5U1	Dynein heavy chain axonemal	18	481.1	5.67

**Fig. 2:**
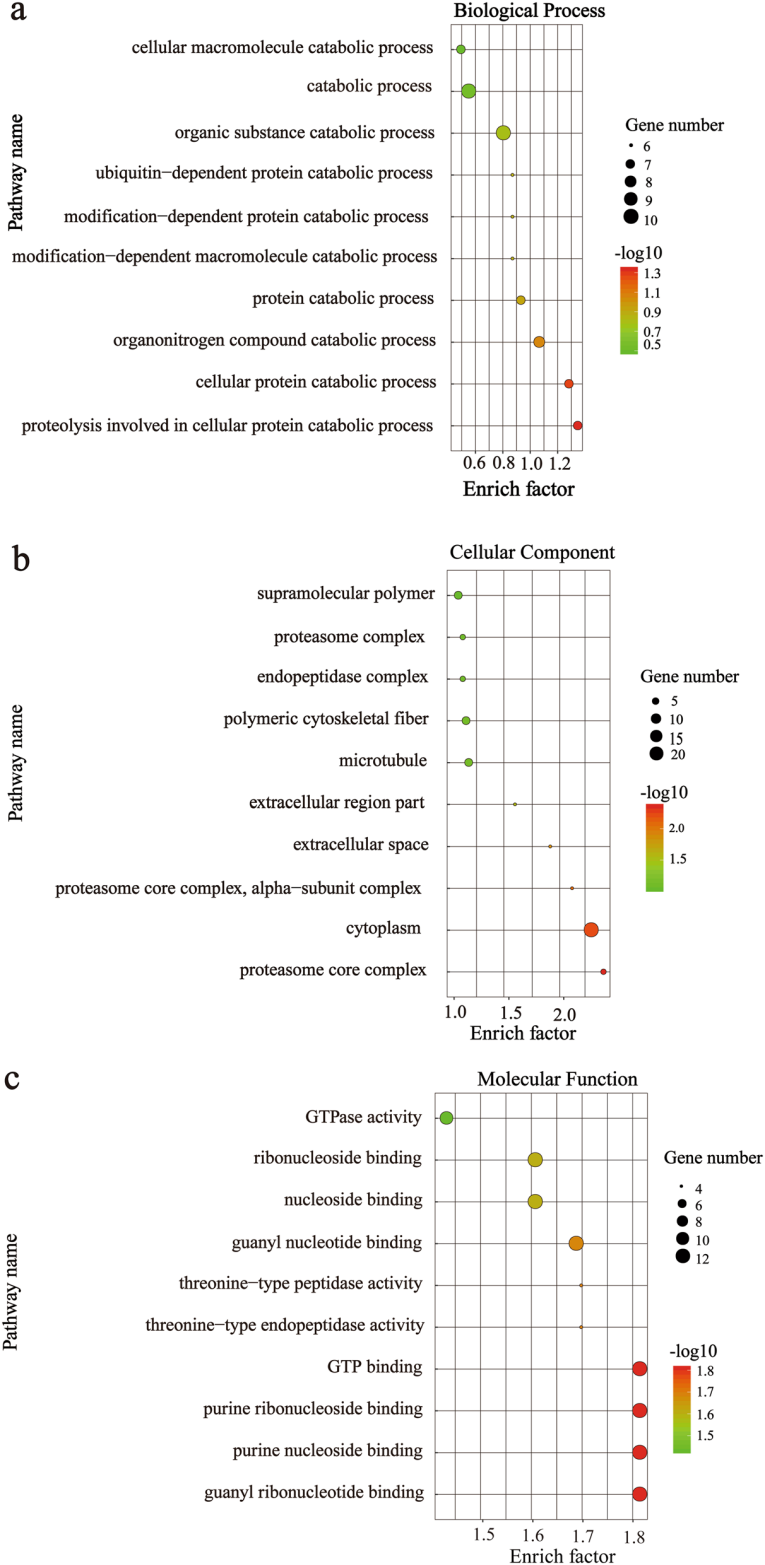
Gene ontology (GO) of protein cargo enriched in *F. gigantica*-derived 100k EVs. **a** GO biological process terms of adult *F. gigantica*-derived 100k EVs, **b** GO molecular function terms of adult *F. gigantica*-derived 100k EVs, **c** GO cellular component terms of adult *F. gigantica*-derived 100k EVs. The size and color of the circle represent the number of genes and the *P*-value

Following a gene ontology (GO) analysis, the most represented GO terms in the category biological processes in the *F. gigantica* 100k EV proteins were assigned as “proteolysis involved in cellular protein catabolic process,” “cellular protein catabolic process” and “organonitrogen compound catabolic process” (Fig. 2a). Similarly, the most represented GO terms within the category molecular function were “guanyl ribonucleotide binding,” “purine nucleoside binding,” “purine ribonucleoside binding” and “GTP binding” (Fig. 2b). The “proteasome core complex,” “cytoplasm,” “proteasome core complex,” “alpha-subunit complex” and “extracellular space” terms were classified within the category “cellular component” (Fig. 2c).

### Worm recoveries and percentages of protection

**Fig. 3:**
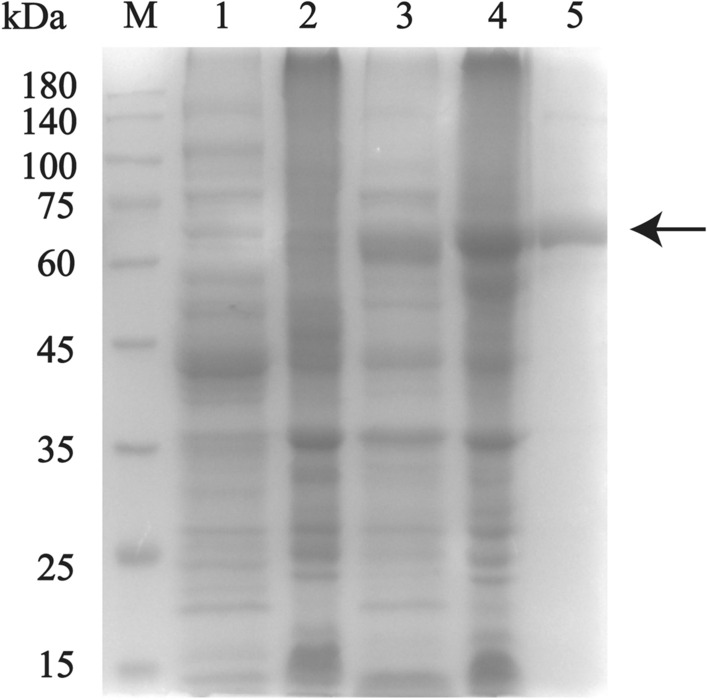
Expression and purification of recombinant* F. gigantica* heat shock protein 70 (rFg-HSP70). Lanes: M, protein marker; 1, supernatant expression products of pET-28a induced by isopropylthio-β-galactoside (IPTG); 2, inclusion body of pET-28a induced by IPTG expression products; 3, supernatant expression products of pET-28a-FgHSP70 induced by IPTG; 4, 5, purification of rFg-HSP70 (arrow)

**Fig. 4:**
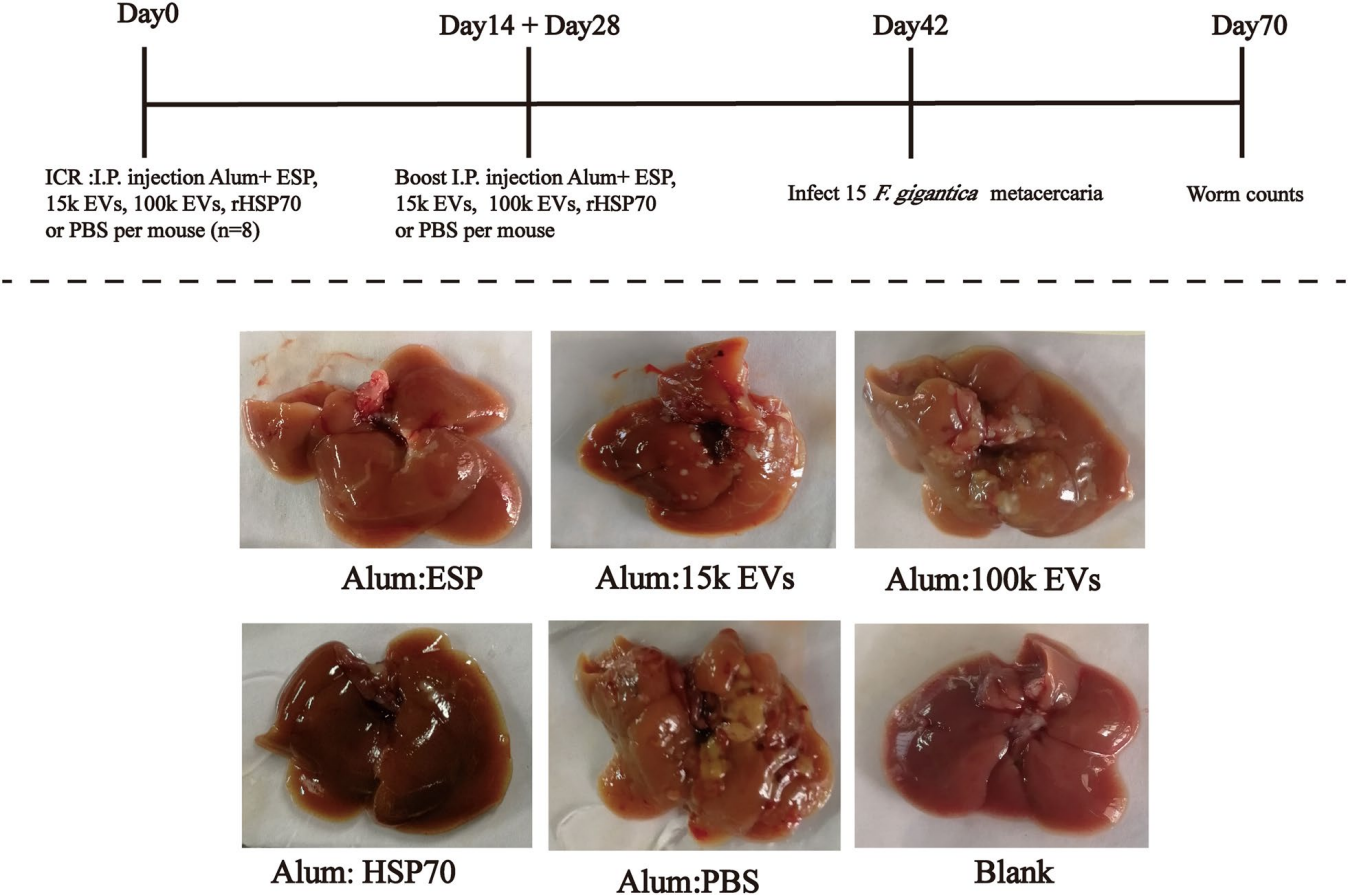
EVs stimulate protective immunity against *F. gigantica* metacercariae challenge in ICR mice. Female ICR mice, *n* = 8 per group, were vaccinated (intraperitoneally [I.P.]) with 15k EVs, 100k EVs, ESPs, rFg-HSP70 or PBS in alum adjuvant prior to challenge with 15 *F. gigantica* metacercariae. Shown are representative pictures of macroscopic pathological lesions in livers of infected mice recovered at 4 weeks post-infection. Groups: Alum:ESP, ESP-immunized and infected group; Alum:15k EVs, 15k EV-immunized and infected group; Alum:100k EVs, 100k EV-immunized and infected group; Alum:HSP70, rFg-HSP70-immunized and infected group; Alum:PBS, adjuvant-infected control group; Blank, non-immunized and non-infected group. ESPs, Excretory/secretory products

**Fig. 5:**
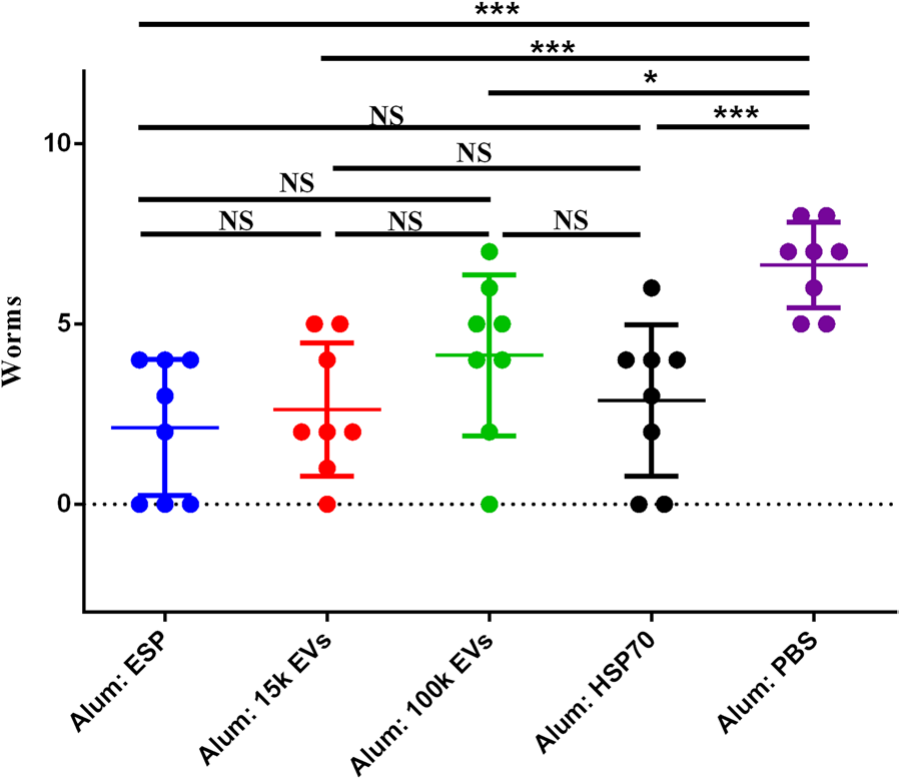
Fluke burden from each ICR mouse was recorded at 28 days post-infection (*n* = 8). Asterisks indicate level of significance between groups: *P < 0.05, **P< 0.01, ***P < 0.001. NS, Non-significant

Proteomic data indicated that many of the proteins present in the *F. gigantica*-secreted 100k EVs are known vaccine candidates. We tested whether immunization with *F. gigantica* ESPs (depleted of EVs), 15k EVs, 100k EVs and rFg-HSP70 (Fig. 3) could induce protective immunity in vivo. Following vaccination and subsequent *F. gigantica* metacercariae challenge, gross liver lesions appeared which were similar in all infected mice. These lesions were characterized by fibrosis spots, tortuous whitish tracts and patches and thickened and calcified deposits on the surface (Fig. 4). Representative liver lesion area reduction was observed in mice after vacination with ESPs, 15k EVs, 100k EVs and rFg-HSP70 vaccination (Fig. 4). The mice immunized with 100k EVs, 15k EVs and ESPs also showed a marked reduction in fluke burden compared with adjuvant control groups. The percentages of protection were 37.73% (one-way ANOVA,* F*_(4, 35)_ = 7.345, *P* = 0.0402) for *F. gigantica* 100k EVs, 60.38% (one-way ANOVA,* F*_(4, 35)_ = 7.345, *P* = 0.0006) for *F. gigantica* 15k EVs, 67.90% (one-way ANOVA,* F*_(4, 35)_ = 7.345, *P* = 0.0001) for *F. gigantica* ESPs and 56.6% for rFg-HSP 70 (one-way ANOVA, * F*_(4, 35)_ = 7.345, *P* = 0.0013) (Fig. 5).

### Antibody levels

**Fig. 6:**
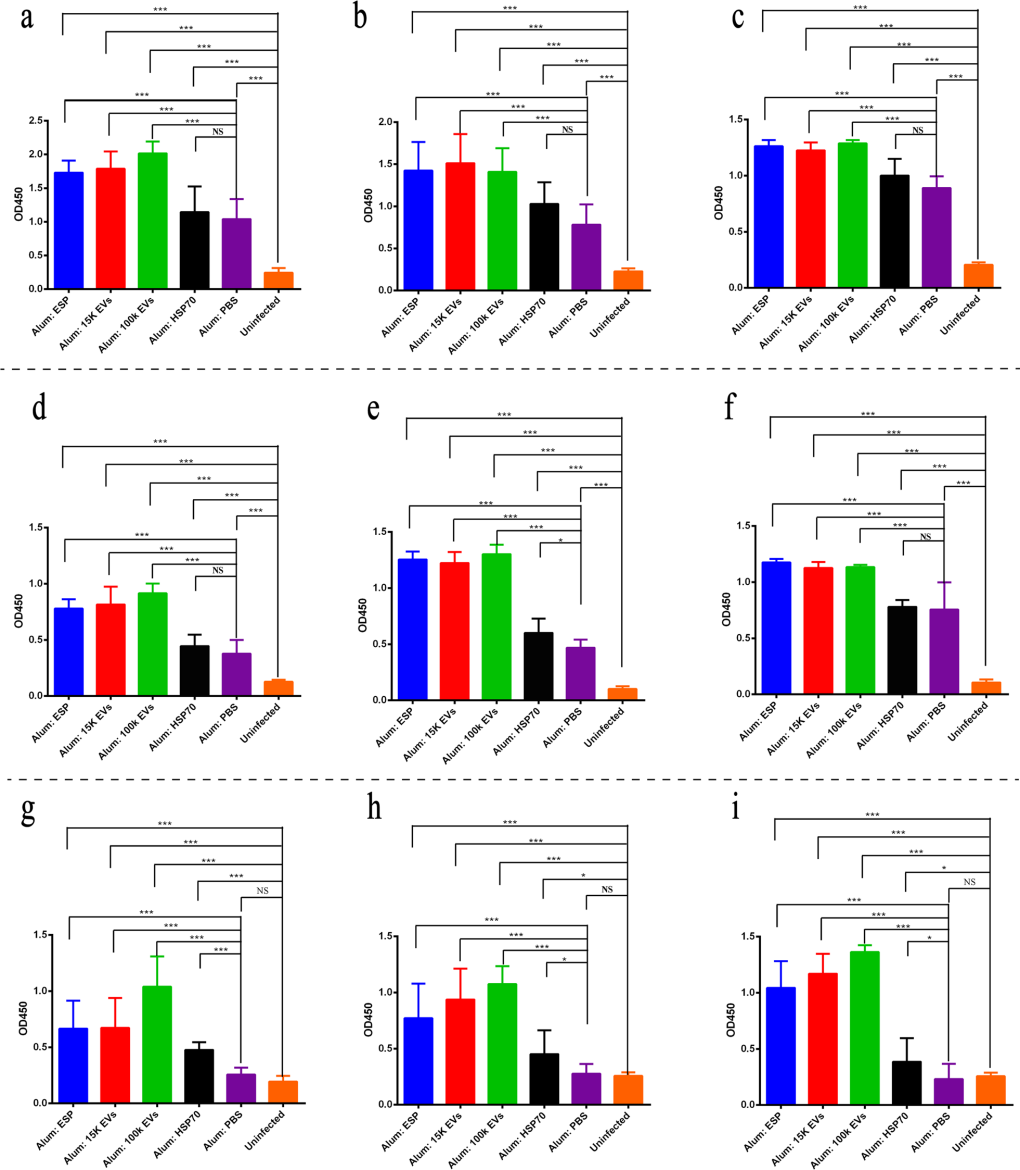
Serum IgG, IgG1, IgG2a levels in vaccinated ICR mouse. Serum IgG (**a**–**c**), IgG1(**d**–**f**) and IgG2a (**g**–**i**) levels were measured from the vaccinated groups (mice immunized with 15k EVs, 100k EVs, ESPs and PBS in alum adjuvant prior to challenge with 15 *F. gigantica* metacercariae) and non-immunized and uninfected group. Mean IgG (**a**–**c**), IgG1 **(d**–**f**) and IgG2a (**g**–**i**) levels were measured against ESPs (**a**, **d**, **g**), 15k EVs (**b**, **e**, **h**) and 100k EVs (**c**, **f**, **i**) by indirect enzyme-linked immunosorbent assay. Results represent the mean absorbance measured at 450 nm for each group. Asterisks indicate level of significance between groups: *P < 0.05, **P < 0.01, ***P < 0.001. NS, Non-significant

Anti-parasite-specific IgG1 and IgG2a serum antibodies are often used as reference indexes of resistance or chronicity during *F. gigantica* infection [26, 33]. ICR mice vaccinated with 15k EVs, 100k EVs and ESP showed a significant increase in sera-specific IgG (Fig. 6a–c), IgG1 (Fig. 6d–f) and IgG2 (Fig. 6g–i) levels compared with the adjuvant control group and the blank group. Mice immunized with 100k EVs produced distinct levels of 15k EVs and ESP-responsive IgG, IgG1 and IgG2. Similarly, sera from mice vaccinated with 15k EVs or *F. gigantica* ESPs contained both IgG, IgG1 and IgG2 reactive to *F. gigantica* 15k EVs, *F. gigantica* 100k EVs and *F. gigantica* ESPs, indicating that they may share homologous protein components. However, there was no significant difference in specific IgG2a between the adjuvant control group and the uninfected group. IgG2a can represent the Th1-type immune response to a certain extent, and induction of the Th1-type immune response and a high level of IgG2a is key to vaccine protection.

## Discussion

The TEM analysis and nanoparticle tracking analysis indicated the successful isolation of two subpopulations of EVs from adult *F. gigantica* culture supernatants using differential centrifugation. Of these, 15k EVs ranged from 91.28 to 825 nm in diameter, and the majority (> 200 nm) had characteristics typical of microvesicles [35], and the 100k EVs ranged from 43.82 to 396.1 nm in diameter and were mainly < 200 nm in diameter, with typical cup-shaped structures. These characteristics are similar to those recorded in our previous studies *F. gigantica* 110k EVs [36] and previously reported 120k exosome-like vesicles isolated from *F. hepatica* [37]. The size and characteristics of the 100k EVs are comparable to exosome-like vesicles isolated from other trematodes, nematodes and cestodes. The dimensions of exosome-like vesicles obtained from *F. hepatica* ranged from 37 to 153 nm [37]. In *Schistosoma mansoni*, the comparable values were 50–130 nm [38]; in *O. viverrini*, 40–180 nm [15]; in *Nippostrongylus brasiliensis*, 60–160 nm [39]; in *Trichuris muris* 30–150 nm [40]; in *Brugia malayi*, 85–200 nm [41]; in *Ascaris suum*, 80–200 nm [42] and in *E. granulosus*, 50– 350 nm [43]. NTA analysis showed that *F. hepatica* 120k EVs and *F. gigantica* 110k EVs are smaller than the 100k EVs [11]. We suggest that centrifugation at 100,000 *g* may result in the loss of some small exosome-like EVs. Exosome biogenesis or vesicle trafficking proteins, ESCRT pathway proteins as well as exosome markers, heat shock proteins and 14-3-3 were identified in 100k EVs during the proteomics analysis. These results indicate successful isolation of exosome-like vesicles from adult *F. gigantica* culture supernatants.

We identified several proteases, such as legumain, cathepsin Bs, cathepsin Ls, proteasome subunit alpha, proteasome subunit beta, proteasome subunit alpha type, aminopeptidase, serpin and cystatin. Proteases produced by helminths are essential for successful tissue invasion during migration [44, 45], the degradation of hemoglobin for food processing, the degradation of IgG for preventing the antibody-dependent cell cytotoxicity response (ADCC) [46], the degradation of immune cell surface receptors [47] and intracellular signaling [48] for immune modulators. We also identified glycolytic enzymes in 100k EVs, including triose phosphate isomerase, GAPDH, phosphoglycerate kinase, phosphoglycerate mutase and glucose-6-phosphate isomerase. Glycolytic enzymes are involved in generating ATP and NADH, which enable helminths to obtain energy [49]. In addition to their role in glycolysis, glycolytic enzymes may function in a variety of other biological functions, such as immune invasion, virulence and immunoregulation [50]. Veamurthy et al. [51] found that GAPDH secreted by *Haemonchus contortus* could bind to complement C3, which may inhibit complement activation and membrane attack complex formation, thus causing immune suppression. GAPDH expressed on the surface of *Plasmodium sporozoite* binds to the CD68 receptor on the surface of Kupffer cells, promoting sporozoite liver invasion [52]. These observations indicate that glycolytic enzymes in 100k EVs are possible vaccine candidates against fascioliasis. The GAPDH of *O. volvulus* [53]*, S. mansoni* [54] and *E. multilocularis* [55] have been evaluated as vaccines with high efficacies.

In *F. gigantica* 100k EVs, some proteins that can bind to *F. gigantica*-positive serum from infected buffaloes were also found, including tubulin alpha, glucose transporter-2 protein, glutathione* S*-transferase, phosphoglycerate kinase, cathepsin B5, cathepsin L, calcium-binding protein, HSP70, kunitz, 14-3-3 protein, leucine amino peptidase, multidomain cystatin, secreted saposin-like protein SAP-3 and thioredoxin [56]. Liu et al. [57] found that *F. hepatica* ESPs could bind with Th1-related cytokines (IL-2 and interferon gamma [IFN-γ]) and Th17-related cytokine (IL-17). We also identified these proteins in our proteome analysis. Proteins such as annexin, phosphoenolpyruvate carboxykinase and fructose-bisphosphate aldolase bind to IL-2; phosphatidylinositol-4,5-bisphosphate 4-phosphatase and fructose-bisphosphate aldolase bind to IFN-γ; and glutathione* S*-transferase, severin, annexin, Sh3-containing grb2 protein 1 and malic enzyme bind to IL-17. Such binding suggests that these proteins bind to the host pro-inflammatory cytokines and deactivate these cytokines. This is also an important part of parasite immune evasion. These data suggest that these proteins may be potential diagnostic markers or vaccine candidates.

HSP70 is an EV-rich fraction, and we found a large amount of HSP70 in the proteomic data. HSP70 is a highly conserved molecular chaperone that plays an essential role in homeostasis during host invasion and helps parasites adapt to rapidly changing environments [58, 59]. HSP70 from helminths are considered to be promising vaccine candidates [60, 61]. HS70 can also be used as adjuvant antigenic conjugate to induce specific antigen-specific immunity [62, 63]. However, the extent to which *F. gigantica* HSP70 can induce a protective immunity response remains unclear. Reduction in the fluke burden is a key parameter for evaluating vaccine efficacy after a challenge with *F. gigantica* metacercariae. In this study, we demonstrated that vaccination with *F. gigantica* 100k EVs, 15k EVs, ESPs (depleted of EVs) and rFg-HSP70 can induce protective immunity against a subsequent *F. gigantica* infection. Mice immunized with ESPs, 15k EVs, 100k EVs and rFg-HSP70 showed significant reductions (*P* < 0.05) in fluke burden, which induced a significant level of protection of 67.9%, 60.4%, 37.7%, and 56.6%, respectively. *Fasciola gigantica* ESPs, 15k EVs and 100k EVs induce high levels of the antigen-specific IgG, IgG1, and IgG2a antibodies in mice. It is possible that common proteins may be shared among *F. gigantica* 100k EVs, 15k EVs and ESPs. Similar results were found in studies of *F. hepatica* [11], *H. polygyrus* [21] and *Echinostoma caproni* [64]*,* demonstrating that overlapping proteins were present in EVs and ESPs isolated from these helminths. Antigen-specific responsive IgG2a antibody could also be important for downstream resistance during *F. gigantica* infection. Higher IgG2a antibody responses have been associated with lower fluke burdens in *Fasciola* spp*.* vaccine research [33, 65, 66], suggesting that the Th1 response may be associated with protection against fascioliasis.

## Conclusion

We purified and characterized the proteomic composition of exosome-like vesicles from *F. gigantica* culture media. *Fasciola gigantica* exosome-like vesicles containing proteins may be related to immunomodulatory, immune evasion and virulence. Vaccination with *F. gigantica* exosome-like vesicles protected mice against subsequent infection by *F. gigantica* metacercariae. Our results may provide good insights into novel strategies for the immunotherapy, vaccination, and diagnosis of fascioliasis.

## Supplementary Information

**Additional file 1: Table S1.** List of proteins identified by MS from EVs isolated from adult *F. gigantica *culture supernatants.

## Data Availability

The datasets supporting the findings of this article are included within the paper and its supplementary materials.

## Abbreviations

ADCC: Antibody-dependent cell cytotoxicity response
DCs: Dendritic cells
DTT: Dithiothreitol
ELISA: Enzyme linked immunosorbent assay
ESPs: Excretory/secretory products
EVs: Extracellular vesicles
GAPDH: Glyceraldehyde-3-phosphate dehydrogenase
GO: Gene ontology
HSP70: Heat shock protein 70
IFN-γ: Interferon gamma
IL-2: Interleukin 2
IL-17: Interleukin 17
IPTG: Isopropylthio-β-galactoside
LC–MS/MS: Liquid chromatographytandem mass spectrometry
NTA: Nanoparticle tracking analysis
PBS: Phosphate-buffered saline
SDS-PAGE: Sodium dodecyl sulfate polyacrylamide gel electrophoresis
TEM: Transmission electron microscopy
